# GPs’ recognition of death in the foreseeable future and diagnosis of a fatal condition: a national survey

**DOI:** 10.1186/1471-2296-14-104

**Published:** 2013-07-22

**Authors:** Susanne JJ Claessen, Anneke L Francke, Michael A Echteld, Bart PM Schweitzer, Gé A Donker, Luc Deliens

**Affiliations:** 1 VU University medical center (VUmc), EMGO Institute for Health and Care Research, Department of Public and Occupational Health & Expertise Center Palliative Care VUmc, Amsterdam, the Netherlands; 2 NIVEl, Netherlands Institute for Health Services Research, Utrecht, the Netherlands; 3 Erasmus Medical Center Rotterdam, Intellectual Disability Medicine, Department of General Practice, Rotterdam, the Netherlands; 4 VU University medical center (VUmc), EMGO Institute for Health and Care Research, Department of General Practice, Amsterdam, the Netherlands; 5 Ghent University & Vrije Universiteit Brussel, End-of-life Care Research Group, Brussels, Belgium

**Keywords:** General practice, Primary care, Palliative care, End-of-life care, Diagnosis, Cancer, Chronic diseases

## Abstract

**Background:**

Nowadays, palliative care is considered as a care continuum that may start early in the course of the disease. In order to address the evolving needs of patients for palliative care in time, GPs should be aware in good time of the diagnosis and of the imminence of death. The aim of the study was to gain insight into how long before a non-sudden death the diagnosis of the disease ultimately leading to death is made and on what kind of information the diagnosis is based. In addition, we aimed to explore when, and based on what kind of information, GPs become aware that death of a patient will be in the foreseeable future.

**Methods:**

A written questionnaire focusing on the GPs’ experiences with their last patient who died non-suddenly was sent to a random representative sample of 850 GPs in the Netherlands.

**Results:**

The data were analysed of the 297 GPs who responded. 76% of the reported cases were cancer patients and 24% were patients with another non-sudden cause of death. The diagnosis was made only in the last week of life for 15% of the non-cancer patients and 1% of the patients with cancer. GPs were most likely to have been informed of the diagnosis by the medical specialist, although particularly in the case of non-cancer patients GPs also relied on their own assessment of the diagnosis or on other information sources.

The GP remained unaware that the patient would die in the foreseeable future until the last week of life in 26% of the non-cancer group, while this was the case for only 6% of the cancer patients. GP’s awareness was most likely to be based on the GP’s own observations of problems and/or symptoms.

**Conclusions:**

The GP often only becomes aware of a fatal diagnosis and of death in the foreseeable future at a late stage in the disease trajectory, particularly in the case of non-cancer patients. It can be assumed that if the diagnosis and the nearing death are only recognised at a late stage, palliative care is either started at a very late stage or not at all.

## Background

**Figure 1:**
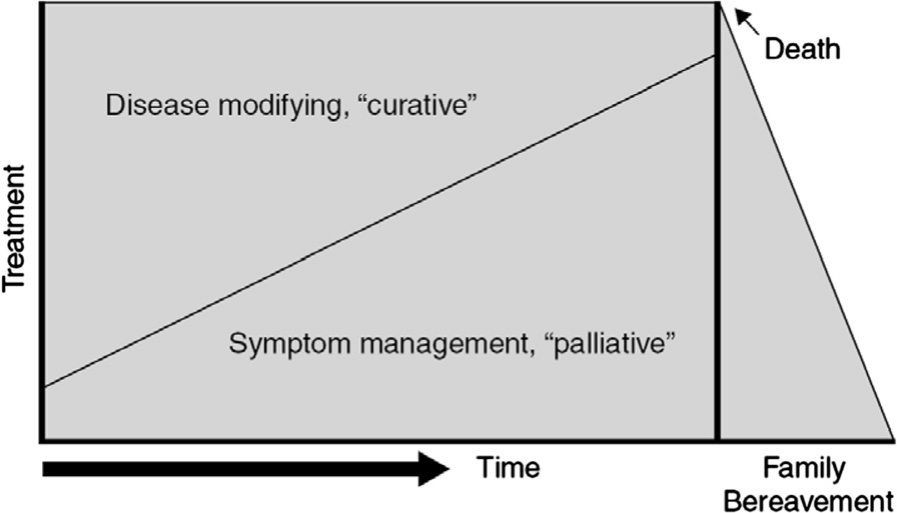
Palliative care as a care continuum (Lynn and Adamson 2003).

Nowadays, care providers, policymakers and researchers are increasingly aware that palliative care is broader than terminal care. Figure 1 shows the ‘model’ of Lynn and Adamson (2003), displaying palliative care as part of a care continuum that starts early in the trajectory of a chronic illness and that ends with the death of the patient and aftercare for relatives [1].

Timely diagnosis of a life-threatening disease as well as timely awareness that death is near are important in enabling anticipatory measures to be taken to cope with the frequent increase in symptoms and problems, in enabling crises to be prevented and in allowing patients’ needs for palliative care to be met fully. In addition, if a GP becomes aware of a fatal diagnosis and of death in the foreseeable future only in a very late stage of the disease, this impedes a timely start to end-of-life care (‘palliative care in the last 12 months of the illness’, [2]). Because the GP is a key professional in palliative care [3,4], it is important that the GP is aware in good time of a diagnosis of a life-threatening condition and of the prognosis that a patient will die in the foreseeable future.

There is literature available about the care of people who died from malignancies, heart failure, respiratory diseases or renal failure [5-7]. However, to our knowledge, no previous studies have been conducted on what kind of information GPs use and in what phase of the illness trajectory GPs become aware of the diagnosis. Yet some studies have been conducted on how professionals identify that a patient will die in the foreseeable future [8,9]. Sullivan *et al*. performed secondary analyses of interviews with hospital physicians [9]. These authors found that 38% of the physicians were uncertain when the patient was admitted whether the patient would die during this hospitalisation. However, over the course of hospitalisation 86% reported that they knew that death was imminent. Eleven percent of physicians reported anticipating the patient would die weeks before the death, 57.1% days before, and 18.3% hours before the death. Earlier recognition of imminent death was associated with greater reported overall satisfaction of the physicians with the end-of-life care provided to the patient. Furthermore, Abarshi *et al*. investigated how long before death GPs recognise that patients were likely to die in the near future [8]. They reported that GPs never recognised that the patient would die in the foreseeable future in about a third of their patients with a non-sudden death. However, the study of Abarshi *et al*. did not explore which sources of information the GPs used to identify death in the foreseeable future.

In addition, so far it has remained unclear whether there are differences between cancer patients and other patients with a non-sudden death cause regarding the ‘when and how’ of GPs’ identification of a fatal diagnosis and of death in the foreseeable future. However, common disease trajectories differ between patient groups [6,10,11] which may also have consequences for GPs’ awareness of the diagnosis and the awareness that the patient's death will be in the foreseeable future. Lynn and Adamson and also Murray distinguished three common disease trajectories leading to a non-sudden death [1,12]. First there is the common illness trajectory of patients with cancer, which is reasonably predictable and usually characterised by a clear terminal phase. In contrast, trajectories in patients with COPD or heart failure are often characterised by intermittent exacerbations and remissions and a relatively sudden death. In the frail elderly, such as people with dementia, there is often a prolonged gradual decline towards death.

Since disease trajectories vary, it can be expected that the timing of the GPs' recognition of the fatal diagnosis and of the awareness that the patient would die in the foreseeable future are also different.

We conducted this study among a random sample of GPs in the Netherlands in order to gain more insight into the ‘how and when’ of GPs’ identification of a fatal diagnosis and of the foreseen death. The following research questions are addressed:

1 How long before death is the diagnosis of a disease ultimately leading to death made in cancer patients and in patients with another non-sudden cause of death?

2 On the basis of what kind of information sources do GPs become aware of the diagnosis of the disease leading to death?

3 How long before the death are GPs aware that death will be in the foreseeable future in cancer patients versus patients with another non-sudden cause of death?

4 On the basis of what kind of information sources do GPs become aware that a patient will die in the foreseeable future?

## Methods

### Design

A retrospective cross-sectional design was used based on survey data.

### Study population and setting

A random sample of 850 Dutch GPs participated in this survey. The sample was drawn from a national registration base (NIVEL) with the addresses and background characteristics of all GPs working in the Netherlands.

### Pilot and content of the questionnaire

The content of the questionnaire was largely based on existing questionnaires: in particular a questionnaire about end-of life care by GPs [13-15] and a registration form used by GPs participating in the Dutch Sentinel General Practice Network [16]. GPs were asked to base their answers on the case of their last patient with a non-sudden death. We explained in the questionnaire that this could be a patient who died of cancer, heart failure, COPD, stroke, dementia, other chronic diseases or “gradual decline because of frailty and old age”.

The questions used for answering the research questions addressed in this paper are shown in Additional file 1.

Face validity, content validity and comprehensibility of the draft questionnaire were assessed by the steering committee, which included three scientists in the field of end-of-life care and two GPs. The usability and comprehensibility were tested further among ten other GPs. This resulted in some minor revisions, e.g. regarding the time period to which questions relate. The final version of the questionnaire consisted of 44 semi-structured questions.

The random sample of 850 GPs received the final questionnaire, together with an explanatory letter and return envelope, in the summer of 2010. Reminders were sent after four weeks and seven weeks.

### Statistical analysis

All questionnaires were scrutinised for errors and missing data, and the data were digitised by scanning. A random sample of 15 questionnaires was checked for errors arising during scanning. No errors were found.

Descriptive analyses and Chi-square analyses were used to answer the research questions addressed in this paper. A Fisher exact test was used instead of a Chi-square test if the expected value for one or more of the cells was less than five.

### Ethics

GPs received information about the aim and content of the research in an explanatory letter, which was sent together with the questionnaire. The anonymity of the GPs and their patients was strictly preserved throughout the data entry and analysis process.

According to Dutch law, no approval of a Medical Ethics Committee is needed for surveys among care professionals and for post-mortem anonymous patient data.

## Results

### Response

Seventeen questionnaires were returned as ‘undeliverable’ (mainly because the address was unknown or incorrect), four other questionnaires were returned uncompleted because the GP was absent due to long-term illness or maternity leave and eleven were returned uncompleted because the GP had no experience with palliative care. Hence, 818 of the 850 GPs in the sample were considered eligible for this study. A total of 297 questionnaires were completed and returned (the net response rate was 36%, i.e. 297/818). About half of the respondents (47%) were aged between 40 and 54. The majority were male (57%) and 86% worked in a two-person or group practice (Table 1).

**Table 1:** Characteristics of sample of GPs in percentages

	**Sample**		
	**Non-respondents (n = 521)**	**Respondents (n = 297)**	P-value*
Gender			0.717
Male	58.5	57.2	
Female	41.5	42.8	
Age			0.720
25–39	25.0	27.0	
40–54	46.8	47.2	
≥ 55	28.2	25.9	
Solo practice			0.169
Yes	17.9	14.1	
No	82.1	85.9	
Degree of urbanisation			0.787
Extremely urbanised/strongly urbanised	47.2	45.1	
Moderately urbanised	18.8	20.5	
Hardly urbanised/not urbanised	34.0	34.4	

*Chi-square analysis.

No differences between non-respondents and respondents were observed regarding gender, age, whether or not the GP had a solo practice, and degree of urbanisation (Table 1).

### Characteristics of the deceased patients dealt with in the GPs’ questionnaires

Of the 297 patients described by the GPs as being their last patient with a non-sudden death, 153 were female (52%). The median age at death was 72 (range: 39–103). The majority (76%) died from cancer, while 24% had another non-sudden cause of death such as heart failure (33%), general decline because of old age (27%), asthma/COPD (9%), dementia (6%), amyotrophic lateral sclerosis (4%), renal failure (4%), stroke (1%), other (11%), and an unknown non-cancer cause of death (6%).

70% of the patients died at home, 13% in a care home and 10% of the patients died in a hospital.

### Time between the diagnosis of the disease ultimately leading to death and the death

The diagnosis of the disease leading to the non-sudden death was only made in the last six months of life in 41% of the group with cancer and 49% of the non-cancer group (see Table 2, difference is not significant). The diagnosis was only made in the last month before death for 29% of the non-cancer patients versus 6% of the cancer patients (p < 0.001). The diagnosis was only made in the last week before death in 15% of the non-cancer patients versus 1% of the cancer patients (p < 0.001).

**Table 2:** Timing of the diagnosis of the disease ultimately leading to death and sources of information

	**Cancer**	**Non-cancer**	
**Timing of diagnosis**	n = 220**	n = 65**	P-value*
	**(%)**	**(%)**	
Not until last year before death (also including last six months, last month and last week)	120 (54)	39 (60)	0.437
Not until last six months before death (also including last month and last week)	90 (41)	32 (49)	0.234
Not until last month before death (also including last week)	14 (6)	19 (29)	**<0.001**
Not until last week before death	1 (1)	10 (15)	**<0.001**
**GPs’ information about diagnosis was based on: (more than one answer possible)**	**Cancer**	**Non-cancer**	
	**n = 226**	**n = 71**	
	**(%)**	**(%)**	
Information from the medical specialist	165 (73)	43 (61)	**0.046**
GPs’ own diagnostics	80 (35)	35 (49)	**0.036**
Information from the patient	34 (15)	5 (7)	0.082
Information from relatives	7 (3)	9 (13)	**0.004**
Other	8 (4)	1 (1)	0.692
Don’t know	1 (0.4)	0 (0)	1.000
Not applicable	1 (0.4)	2 (3)	0.143

*Chi-square analyses: significant differences between group with cancer and non-cancer group are in bold.

**12 missing values, including ‘don’t know/not applicable’ answers.

In 73% of the patients who died from cancer and 61% of the non-cancer patients (p < 0.05) GPs knew the diagnosis through information from the medical specialist - whether or not combined with other sources of information (Table 2). Particularly in non-cancer patients, GPs (also) relied on their own diagnostics (in 49% of the non-cancer patients versus 35% of the cancer patients, p < 0.05). Information from relatives was also more often a source of information in non-cancer patients (13%) than in cancer patients (3%, p < 0.01). In addition, GPs sometimes received information from the patients themselves about the diagnosis (see Table 2).

### Timing of GPs’ awareness of death in the foreseeable future and sources of information

In 86% of the group with cancer and 94% of the non-cancer group, GPs’ awareness that the patient would die in the foreseeable future was at some point in the last six months of life. GPs recognized that death would be in the foreseeable future only in the last week before the decease in 26% of the patients with a non-cancer death cause versus in 6% of the cancer patients (see Table 3, p < 0.001).

**Table 3:** Timing of GPs’ awareness that the patient would die in the foreseeable future and sources of information

	**Cancer**	**Non-cancer**	
**Time between GPs’ awareness and actual death**	n = 221**	n = 68**	P-value*
	**(%)**	**(%)**	
Not until the last year before death (also including last six months, last month and last week)	207 (94)	65 (96)	0.770
Not until the last six months before death (including the last month and last week)	190 (86)	64 (94)	0.072
Not until the last month before death (including the last week)	66 (30)	41 (60)	**<0.001**
Not until the last week before death	13 (6)	18 (26)	**<0.001**
**GPs’ awareness was based on: (more than one answer possible)**	**Cancer**	**Non-cancer**	
	**n = 225**	**n = 71**	
	**(%)**	**(%)**	
GPs’ observation of problems and/or symptoms	175 (78)	62 (87)	0.079
Information from medical specialist	120 (53)	20 (28)	**<0.001**
Information from home-care professionals	6 (3)	9 (13)	**0.002**
Information from relatives	33 (15)	19 (27)	**0.020**
Other	15 (7)	7 (10)	0.371
Not applicable	0 (0)	1 (1)	0.240

*Chi-square analyses: statistically significant differences (P < 0.05) between cancer and non-cancer group are in bold.

**8 missing values, including ‘don’t know’ answers.

GPs’ own observations of problems and/or symptoms made them aware that the patient would die in the foreseeable future in 78% of the cancer patients versus 87% of the patients with another non-sudden cause of death. Information from the medical specialist led to GPs’ awareness of death in the foreseeable future in 53% of the cancer patients versus 28% of non-cancer patients (p < 0.001). Particularly in the non-cancer group, GPs recognized that patients' death would be in the foreseeable future on the basis of information from home-care professionals and/or relatives (significant differences between cancer and non-cancer patients, see Table 3).

## Discussion and conclusions

This study shows that for the majority of cancer patients (99%) and non-cancer patients (85%), the diagnosis of the disease ultimately leading to death was made before the last week of life. However this also means that the diagnosis was not made until the last week of life in 15% of the non-cancer group. Apparently, it is more difficult to make the diagnosis for patients with a non-cancer disease than for patients with cancer. The GPs’ knowledge about the diagnosis was often based on multiple sources of information. For the majority of patients, GPs learnt about the diagnosis through information from the medical specialist. GPs were more likely to rely also entirely or partly on their own diagnostics or information from relatives in the case of patients with diseases other than cancer.

In addition, our results show that GPs sometimes only became aware that the patient would die in the foreseeable future late in the disease trajectory. The GP remained unaware that death would be in the foreseeable future until the last week before death in a quarter of the non-cancer group, while this was the case for only 6% in the group of cancer patients. This may be related to the fact that there is no clear diagnosis for some patients, such as the frail elderly with a general decline towards death. Another explanation may be that in the case of patients with COPD or chronic heart failure, for instance, the medical specialist has the main responsibility for the medical treatment of the patient until a late stage in the disease trajectory. This may be different in other countries and therefore findings cannot automatically generalized. If communication between the GP and medical specialists is poor, the patient’s diagnosis may long be unknown to the GP, which hampers a timely start of palliative care provided by the GP.

The fact that GPs were asked to select their last patient with a non-sudden death may be related to the fact that GPs selected a relatively large number of cancer patients. Van der Velden reported in a death certificate study that about 77,000 people a year die from a chronic disease in the Netherlands. Just over half, 40,000 (52%), die from cancer [17]. In our study, 76% of the patients with a non-sudden death selected by GPs had cancer and 24% were non-cancer patients. Apparently, GPs associate a non-sudden death more with cancer than with a non-cancer disease. The relatively low proportion in our study of patients with stroke (1%) or dementia (6%) is particularly striking. One possible explanation for this under-representation might be that patients with stroke or dementia are more likely to die in nursing homes with their own nursing-home physician being responsible for medical care.

### Recommendations

The present study shows that particularly in the case of non-cancer patients, GPs sometimes remain unaware of the diagnosis of the fatal disease until late in the disease trajectory. It is possible that the GP sometimes only learns of the diagnosis at a very late stage because the medical specialist in the hospital is failing to provide the GP with information. However, it is also possible that a patient has a known fatal illness (e.g. advanced heart failure or COPD) but ultimately dies from another cause (e.g. pneumonia) that has occurred only in the last week before death. Hence, further research is needed to get more insight into the reasons for the finding that the diagnosis of the disease leading to death is only known at such a late stage in a relatively large group of patients who died non-suddenly.

However, in general GPs’ late recognition of the fatal diagnosis and of death in the foreseeable future may have consequences for advance care planning and timely anticipation of the evolving symptoms and care needs of patients. In line with Fitzsimons *et al*. [5], we would like to point out the necessity of embracing the palliative care approach at an early stage of the disease in order to address the evolving needs of patients with a life-threatening chronic illness in good time. Future research is recommended on the disease trajectories from the diagnosis until the death of patients with chronic progressive diseases such as heart failure and COPD.

We also recommend a proactive attitude from GPs in patients with progressive, ultimately terminal diseases. From other recent research it is known that Dutch GPs in general have a reactive, rather than a proactive, attitude in the interactions with their patients [18]. GPs consider it important for a patient to indicate what support he or she needs and they do not want to patronise the patient or give care that is not needed. However, at the end of life a more proactive approach, e.g. involving initiatives by the GP for advance care planning, may result in better matching of patients’ and family members’ existing and evolving care needs.

### Strengths and limitations

A strength of this study is that data are included about both cancer patients and patients with other chronic diseases and the frail elderly. Previous studies of palliative care have mainly focused on cancer patients [19]. However, the net response rate for the GP questionnaire was not high (36%), although comparable with other recent surveys among Dutch GPs [20,21]. It is known that Dutch GPs have a high workload [22], which may explain why the non-response in this group is often high. It could be that GPs with a specific interest in palliative care were more likely to respond, which may have led to overestimation of the GP’s role in making the diagnosis and the identification of patients' death in the foreseeable future.

Another limitation of this survey is that it only involved GPs. It would also be interesting to explore the perspectives of medical specialists on making the diagnosis and on the communication about the diagnosis with the GP, patient and family. In addition, nurses or close relatives, for instance, may play an important role in the recognition that the patient will die within a foreseeable period, and are also an important information source for the GP. Future multi-perspective research on making fatal diagnoses and on the identification of patients' death in the foreseeable future is therefore recommended.

## Competing interests

The authors have declared no competing interests.

## Authors’ contributions

SC performed the draft and the statistical analyses of this paper together with AF. ME and LD initiated the study and obtained the funding. AF, BS, GD and LD supervised the project. All authors read and approved the final manuscript.

## Pre-publication history

The pre-publication history for this paper can be accessed here:

http://www.biomedcentral.com/1471-2296/14/104/prepub

## Supplementary Material

Additional file 1 Questions that are used for the analysis in this paper. Click here for file
