# Supplementary material for: GPs’ recognition of death in the foreseeable future and diagnosis of a fatal condition: a national survey
Source: BMC Fam Pract. 2013 Jul 22;14:104. doi: 10.1186/1471-2296-14-104 (PMC3722000; doi:10.1186/1471-2296-14-104)
Supplement: Additional file 1 — Questions that are used for the analysis in this paper. [file 1471-2296-14-104-S1.doc]

Additional file 1: questions that are used for the analysis in this paper

| 1. What was the underlying disease leading to death? Only one answer possible.   ❑ Cancer, namely ❑ lung ❑ bowel ❑ breast ❑ prostate ❑ other, namely…….  ❑ Heart failure  ❑ Asthma/COPD  ❑ Stroke  ❑ Dementia  ❑ Slow decline because of old age  ❑ Other, namely………………………………………...  ❑ Unknown   1. How long before death was the diagnosis made of the disease that ultimately led to the patient’s death?   ❑ ≤ 7 days before death  ❑ 1-4 weeks before death  ❑ 1-3 months before death  ❑ 4-6 months before death  ❑ 6-12 months before death  ❑ 1-2 years before death  ❑ More than 2 years before death  ❑ I don’t know  ❑ Not applicable because……………………………….   1. How did you become aware of the diagnosis? Multiple answers possible.   ❑ Information from the medical specialist(s)  ❑ Own diagnostics  ❑ Patient him/herself  ❑ Relative(s)  ❑ Other, namely………………………………………...  ❑ I don’t know  ❑ Not applicable because……………………………….   1. How long before death did you become aware of the prognosis that this patient would die in the foreseeable future?   ❑ ≤ 7 days before death  ❑ 1-4 weeks before death  ❑ 1-3 months before death  ❑ 4-6 months before death  ❑ 6-12 months before death  ❑ 1-2 years before death  ❑ More than 2 years before death  ❑ I don’t know   1. How did you become aware of the prognosis that this patient would die in the foreseeable future? Multiple answers possible   ❑ Through problems and/or symptoms that the patient had and that I myself identified as the GP  ❑ Through information from the medical specialist(s)  ❑ Through information from home-care professionals  ❑ Through information from the patient’s relative(s)  ❑ Other, namely…………………………………………  ❑ Not applicable because……………………………….. |
| --- |
